# Rationale and design of an independent randomised controlled trial evaluating the effectiveness of aripiprazole or haloperidol in combination with clozapine for treatment-resistant schizophrenia

**DOI:** 10.1186/1745-6215-10-31

**Published:** 2009-05-15

**Authors:** Michela Nosè, Simone Accordini, Paola Artioli, Francesco Barale, Corrado Barbui, Rossella Beneduce, Domenico Berardi, Gerardo Bertolazzi, Bruno Biancosino, Alfredo Bisogno, Raffaella Bivi, Filippo Bogetto, Marianna Boso, Alberto Bozzani, Piera Bucolo, Marcello Casale, Liliana Cascone, Luisa Ciammella, Alessia Cicolini, Gabriele Cipresso, Andrea Cipriani, Paola Colombo, Barbara Dal Santo, Michele De Francesco, Giorgio Di Lorenzo, Walter Di Munzio, Giuseppe Ducci, Arcadio Erlicher, Eleonora Esposito, Luigi Ferrannini, Farida Ferrato, Antonio Ferro, Nicoletta Fragomeno, Vincenzo Fricchione Parise, Maria Frova, Francesco Gardellin, Nicola Garzotto, Andrea Giambartolomei, Giancarlo Giupponi, Luigi Grassi, Natalia Grazian, Lorella Grecu, Gualtiero Guerrini, Francesco Laddomada, Ermanna Lazzarin, Camilla Lintas, Francesca Malchiodi, Lara Malvini, Livio Marchiaro, Alessandra Marsilio, Massimo Carlo Mauri, Antonio Mautone, Marco Menchetti, Giuseppe Migliorini, Marco Mollica, Daniele Moretti, Serena Mulè, Stylianos Nicholau, Flavio Nosè, Guglielmo Occhionero, Anna Maria Pacilli, Stefania Pecchioli, Mauro Percudani, Ennio Piantato, Carlo Piazza, Francesco Pontarollo, Roger Pycha, Roberto Quartesan, Luciana Rillosi, Francesco Risso, Raffella Rizzo, Paola Rocca, Stefania Roma, Matteo Rossattini, Giuseppe Rossi, Giovanni Rossi, Alessandra Sala, Claudio Santilli, Giuseppe Saraò, Antonio Sarnicola, Francesca Sartore, Silvio Scarone, Tiziana Sciarma, Alberto Siracusano, Stefania Strizzolo, Michele Tansella, Gino Targa, Annamarie Tasser, Rodolfo Tomasi, Rossana Travaglini, Antonio Veronese, Simona Ziero

**Affiliations:** 1 Department of Medicine and Public Health, Section of Psychiatry and Clinical Psychology, University of Verona, Italy; 2 Unit of Epidemiology and Medical Statistics, Department of Medicine and Public Health University of Verona, Italy; 3 Dipartimento di Salute Mentale, ASL n. 3 " Genovese", Genova, Italy; 4 UOP 51 Psichiatria A.O. San Paolo, Milano, Italy; 5 Department of Applied Health and Behavioral Sciences, Section of Psychiatry, University of Pavia, Italy; 6 Centro S. Giovanni di Dio IRCCS FBF, Brescia, Italy; 7 Institute of Psychiatry, Bologna University, Italy; 8 Dipartimento Salute Mentale- Servizio Psichiatrico Area sud-ULSS 22, Verona, Italy; 9 Section of Psychiatry, Department of Medical Sciences of Communication and Behaviour, University of Ferrara and Department of Mental Health, Ferrara, Italy; 10 DSM ASL Salerno 1, Salerno, Italy; 11 Dipartimento di Neuroscienze, Universita' degli Studi di Torino, Italy; 12 Dipartimento Salute Mentale, ASL Taranto, Italy; 13 DSM, ASL 19, Asti, Italy; 14 Unità Operativa Salute Mentale, distretto 112/113, ASL Salerno 3, Salerno, Italy; 15 Dipartimento di Salute Mentale, ASL n. 3 " Genovese", Genova, Italy; 16 Psychiatric Unit of Bollate, Department of Mental Health, Hospital "G. Salvini", Garbagnate Milanese, Milano, Italy; 17 CSM, Azienda Sanitaria di Bolzano, Italy; 18 Unità Operativa Complessa di Psichiatria, Dipartimento di Neuroscienze, Facoltà di Medicina e Chirurgia, Università degli Studi di Roma "Tor Vergata", Roma, Italy; 19 UOC, SPDC, Ospedale San Filippo Neri, ASL RM E, Roma, Italy; 20 Azienda Ospedaliera "Ospedale Niguarda Ca' Granda", Milano, Italy; 21 Unità Operativa n42, Rho, Azienda Ospedaliera "G. Salvini", Garbagnate Milanese, Milano, Italy; 22 DSM, ASL n2 "Savonese", Savona, Italy; 23 ASF-Toscana (Centro Salute Mentale del MOM-SMA Q2), Firenze, Italy; 24 U.O.Salute Mentale "Roseto", ASL AV2, Avellino, Italy; 25 Centro di Salute Mentale di Vicenza (ULSS 6), Vicenza, Italy; 26 First Psychiatric Service, ULSS 20, Ospedale Civile Maggiore, Verona, Italy; 27 Centro Salute mentale ASL 5, La Spezia, Italy; 28 Servizio Psichiatrico, AO Melegnano, sede Gorgonzola, Italy; 29 Struttura Complessa di Psichiatria, A.S.L. CN1, Cuneo, Italy; 30 Azienda Ospedaliera "Carlo Poma", Mantova, Italy; 31 Clinical Psychiatry, IRCCS Ospedale Maggiore Policlinico, Milano, Italy; 32 Fourth Psychiatric Service, ULSS 20, San Bonifacio, Verona, Italy; 33 Second Psychiatric Service, ULSS 20, Ospedale Civile Maggiore, Verona, Italy; 34 SPDC c/o Azienda Osp. Naz. Ss Antonio E Biagio-Alessandria, Italy; 35 Servizio Psichiatrico di Brunico (BZ), Azienda Sanitaria di Bolzano, Italy; 36 Sezione di Psichiatria, Psicologia Clinica e Riabilitazione Psichiatrica, Dipartimento di Medicina Clinica e Sperimentale, Università degli Studi di Perugia, Italy

## Abstract

**Background:**

One third to two thirds of people with schizophrenia have persistent psychotic symptoms despite clozapine treatment. Under real-world circumstances, the need to provide effective therapeutic interventions to patients who do not have an optimal response to clozapine has been cited as the most common reason for simultaneously prescribing a second antipsychotic drug in combination treatment strategies. In a clinical area where the pressing need of providing therapeutic answers has progressively increased the occurrence of antipsychotic polypharmacy, despite the lack of robust evidence of its efficacy, we sought to implement a pre-planned protocol where two alternative therapeutic answers are systematically provided and evaluated within the context of a pragmatic, multicentre, independent randomised study.

**Methods/Design:**

The principal clinical question to be answered by the present project is the relative efficacy and tolerability of combination treatment with clozapine plus aripiprazole compared with combination treatment with clozapine plus haloperidol in patients with an incomplete response to treatment with clozapine over an appropriate period of time. This project is a prospective, multicentre, randomized, parallel-group, superiority trial that follow patients over a period of 12 months. Withdrawal from allocated treatment within 3 months is the primary outcome.

**Discussion:**

The implementation of the protocol presented here shows that it is possible to create a network of community psychiatric services that accept the idea of using their everyday clinical practice to produce randomised knowledge. The employed pragmatic attitude allowed to randomly allocate more than 100 individuals, which means that this study is the largest antipsychotic combination trial conducted so far in Western countries. We expect that the current project, by generating evidence on whether it is clinically useful to combine clozapine with aripiprazole rather than with haloperidol, provides physicians with a solid evidence base to be directly applied in the routine care of patients with schizophrenia.

**Trial Registration:**

**Clincaltrials.gov Identifier**: NCT00395915

## Background

Schizophrenia is a disabling mental disorder [1]. It affects as much as 1% of the population worldwide and it is characterised by psychotic symptoms, including delusions and hallucinations, negative symptoms, characterised by "loss of function", and cognitive impairment [2].

A proportion of one fifth to one third of patients with schizophrenia derive little or no benefit from treatment with conventional or novel antipsychotics [3]. In these treatment-refractory patients, e.g. individuals who had not responded, or had intolerable side-effects, to conventional and novel agents, clozapine has been shown to be the treatment of choice [4-6]. Clozapine is, however, only effective in producing clinically significant symptom improvement in 30–50% of people receiving treatment. One third to two thirds of people still have persistent psychotic symptoms despite clozapine monotherapy of adequate dosage, or have unwanted side-effects that do not permit an adequate up titration of clozapine [7].

Under real-world circumstances, the need to provide effective therapeutic interventions to patients who do not have an optimal response to clozapine has been cited as the most common reason for simultaneously prescribing two or more antipsychotic drugs in combination treatment strategies [8]. Similarly, adopting a pragmatic attitude, European and American treatment guidelines recognize that the concurrent prescription of a second antipsychotic in addition to clozapine is a common-sense strategy in these partially responsive patients [9-12]. However, it remains unclear if there is an evidence base to support one specific antipsychotic in combination with clozapine [13-16]. In a clinical area where the pressing need of providing therapeutic answers has progressively increased the occurrence of antipsychotic polypharmacy, despite the lack of robust evidence of its efficacy, we sought to implement a pre-planned protocol where two alternative therapeutic answers are systematically provided and evaluated within the context of a pragmatic, multicentre, independent randomised study.

The article reported here aims at providing a description of the following background aspects related to the development and implementation of this project: (a) the Italian legislation on independent trials; (b) the concept of pragmatic trials; (c) the marketing of aripiprazole, a novel antipsychotic drug. We additionally provide a description of the main aspects related to the design and current status of the Clozapine plus Haloperidol or Aripiprazole Trial (CHAT).

### Italian Legislation on Independent Trials

The Italian context of care is an ideal setting for independent randomised trials, given the implementation of a National Law (Decreto Ministeriale 17/12/04) that formally recognised the public health value of independent studies investigating the real-world effectiveness of already marketed pharmacological treatments. In 2004 a Ministerial Decree was issued establishing rules to help implement pragmatic independent phase IV clinical trials. In essence, the Decree states that if the following set of conditions are met, (i) the study coordinating centre is independent of drug company support, (ii) study results can be disseminated autonomously, (iii) there is no personal financial interest in studying the drugs included in the trial, (iv) the study drugs are licensed for the indication to be investigated, then the National Health Service (NHS) materially supports the conduct of the trial in three ways: (i) drug costs are paid by the NHS; (ii) there are no fees for submitting the study protocol to the local Ethics Committees; (iii) continuing medical education credits are provided to local investigators.

Considering that all above mentioned criteria are met by CHAT, we took fully advantage of such legislation. In particular, drug costs (clozapine, aripiprazole and haloperidol) are covered by the local health authorities, with two advantages: first, we had the possibility to carry out this study on a low budget, independently from drug companies and from other agencies; second, the drugs under study are prescribed in a way that is identical to that normally followed under real-word circumstances, with obvious advantages in terms of generalisability of study findings.

### Pragmatic Versus Explanatory Design

In recent years there has been a renewal of interest in pragmatic trials (also called practical, effectiveness or management trials), that is for studies that randomly assign real-world patients to licensed drugs with the aim of assessing their effectiveness [17-20]. While explanatory (or phase III) trials answer questions about whether an intervention can work under ideal conditions (efficacy), pragmatic (or phase IV) trials attempt to answer questions about whether an intervention will work in the real world. Explanatory trials are usually carried out by the pharmaceutical industry, while pragmatic trials are more often undertaken by groups of clinical researchers. Recent examples of pragmatic trials include the Clinical Antipsychotic Trials of Intervention Effectiveness (CATIE) [21] and the Cost Utility of the Latest Antipsychotic Drugs in Schizophrenia Study (CUtLASS) [22].

In Italy a seminal pragmatic study was an unblinded trial of intravenous streptokinase in early acute myocardial infarction that enrolled 11,806 patients in one hundred and seventy-six coronary care units [23]. The first report of this influential study was published in 1986 and in subsequent years there was an ongoing debate about the need to support such research.

In the field of mental health, however, only in very recent years criticism has focused on the current standard of the design of explanatory clinical trials. These studies typically enrol highly selected patients that are shortly followed and assessed with rating scales that are seldom used in clinical practice. In Italy this criticism has progressively led mental health professionals to constitute research networks with the aim of developing pragmatic studies. Such studies, ideally, are intended to answer real-world questions by enrolling everyday patients to be followed in the long-term using pragmatic outcome criteria commonly used in practice. Pragmatic measures include suicide attempts, treatment switching, hospitalization, school failure or truancy, job loss, or treatment discontinuation [17,18,24]. CHAT is the first Italian example of this new attitude [25], and other studies will soon follow [26].

### Aripiprazole, a novel antipsychotic drug

In recent years the availability of newer antipsychotic agents has increased the therapeutic options available in the management of clozapine partial responders and, among these newer agents, anecdotal reports have hypothesised a promising role for aripiprazole [27,28]. Aripiprazole is a potent (high-affinity) partial agonist at D2 and 5-HT1A receptors and a potent antagonist at 5-HT2A receptors. In contrast to some of the other atypical antipsychotic agents, treatment with aripiprazole appears to be associated with minimal weight gain and minimal negative impact on metabolic parameters, a key aspect given that these adverse effects might occur during clozapine treatment [29,30]. In terms of positive symptoms, it has been suggested that the combination of clozapine and aripiprazole may lead to greater D2 receptor antagonism in mesolimbic pathways, and, additionally, may combine D2 and D4 antagonism (although the role of D4 receptors in antipsychotic efficacy is unclear). A challenging neurobiological rationale, with a highly synergistic antipsychotic potency without increasing the risk of adverse effects, has therefore been proposed [15]. Henderson and colleagues, who conducted a six-week open label trial to examine the effects of adjunctive aripiprazole in clozapine-treated subjects, showed that this combination had little or no effect in terms of psychotic symptoms, but was associated with a significant decrease in weight, body mass index, fasting total serum cholesterol and total triglycerides [31]. The only randomised placebo-controlled trial published so far, which included 62 clozapine-treated patients with refractory schizophrenia that were randomly assigned to double-blind combination treatment with aripiprazole or placebo, showed that aripiprazole did not lead to better control of symptom severity after 8 weeks of treatment, but benefits were observed in terms of negative symptoms [32].

Other trials employed a design similar to that of CHAT, that is pragmatically assessed the relative efficacy of competitive combination strategies, including clozapine + risperidone versus clozapine + sulpiride, clozapine + quetiapine versus clozapine + amisulpiride, and clozapine + risperidone versus clozapine + ziprasidone [33-35].

## Design and Methods

### Design of The Clozapine Haloperidol Aripiprazole Trial (CHAT)

**Figure 1:**
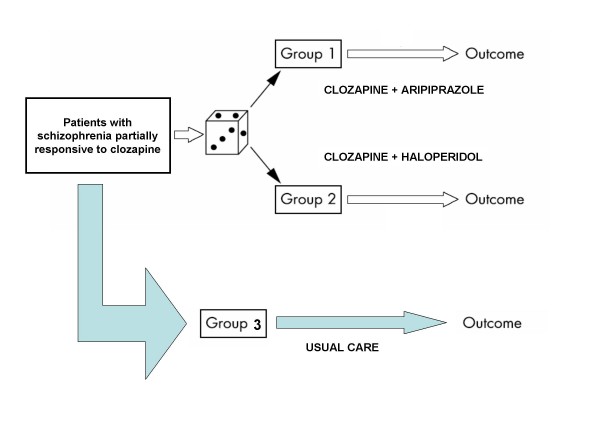
**CHAT study design: randomised and observational cohort**.

**Figure 2:**
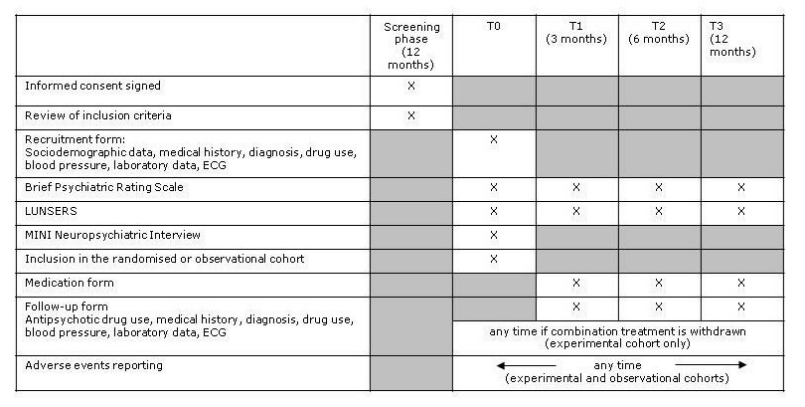
**Study schedule: instruments and forms used at baseline and follow up-interviews**.

The principal clinical question to be answered by CHAT is the relative effectiveness and tolerability of combination treatment with clozapine plus aripiprazole compared to combination treatment with clozapine plus haloperidol in patients with an incomplete response to treatment with clozapine over an appropriate period of time. CHAT is a prospective, multicentre, randomized, parallel-group, superiority trial that follows patients over a period of 12 months. Consecutive patients meeting the trial entry criteria were randomly assigned to combination with aripiprazole or haloperidol. These patients constituted the randomised cohort (Figure 1). Patients meeting the trial entry criteria that were not randomly assigned to competitive treatments were followed under real-world circumstances. These patients constituted the observational cohort (Figure 1). In both the experimental and observational cohort patients and clinicians were not blind to pharmacological treatments provided during the trial. Patients will be assessed at baseline, at 3, 6 and 12 months using the instruments reported in Figure 2.

According to Italian legislation, ethics approval was received in each participating site. All phases of CHAT will be recorded following the Consolidated Standard of Reporting of Trials (CONSORT) statement [36].

### Primary outcome

Withdrawal from allocated treatment within 3 months is the primary outcome. This outcome was selected because stopping or changing antipsychotic combination treatment is a frequent occurrence and major problem in the treatment of patients with schizophrenia. In addition, according to Lieberman and colleagues, this measure integrates patients' and clinicians' judgments of efficacy, safety, and tolerability into a global measure of effectiveness that reflects their evaluation of therapeutic benefits in relation to undesirable effects [21].

### Secondary outcomes

Withdrawal from allocated treatment within 12 months, and time to withdrawal, are used to assess the overall acceptability and efficacy over a long period of time. Additionally, severity of illness is measured by means of the Brief Psychiatric Rating Scale (BPRS) [37]. The BPRS consists of 24 items measuring the following dimensions: positive symptoms, negative symptoms, depression/anxiety and disorganization. All investigators received training to use this rating scale. However, no formal inter-rater exercise has been performed.

Instead of measuring adverse events as observed and reported by the treating clinicians, CHAT measured the perspective of patients exposed to antipsychotic agents by means of the Liverpool University Neuroleptic Side-Effect Rating Scale (LUNSERS) [38]. LUNSERS is a self-rated, semi-structured interview consisting of 51 items that produces a total score that indicates the burden of side effects as perceived by patients (subjective tolerability).

### Inclusion/exclusion criteria

Patients were recruited in Italy. Community psychiatric services agreeing to take part to the study were asked to recruit inpatients and outpatients meeting the inclusion/exclusion criteria over a period of 24 months. Patients meeting the eligibility criteria and the criteria for random allocation (see Additional file 1) were randomly allocated to either aripiprazole or haloperidol, in combination with clozapine, and will be followed for 12 months (experimental cohort). Patients meeting the eligibility criteria, but not the criteria for random allocation, will be followed for 12 months under real-world circumstances and assessed using the same assessment tools employed for patients included in the experimental cohort (observational cohort).

### Pharmacological treatments

In the experimental cohort, in order to resemble everyday clinical practice, clinicians were allowed to prescribe the allocated pharmacological treatments (starting dose and dose changes) according to clinical status and circumstances. All dose changes will be recorded. Following randomization, treatment is to be taken daily for 1 year unless some clear reason to stop develops. Before random allocation, patients were asked to discontinue any antipsychotic drugs other than clozapine. Long-acting antipsychotic drugs needed to be discontinued for at least two weeks before random allocation. All other concomitant medications were permitted. Routine care outside the trial continued as usual. During the study, participants are seen as often as clinically indicated with no extra visits required for the trial.

Patients in the observational cohort received pharmacological and non-pharmacological treatment as clinically indicated. In addition, participants are seen as often as clinically indicated with no extra visits required.

### Power analysis for sample size calculation

At the time of development of the CHAT, only one antipsychotic trial employed discontinuation by any cause as the primary endpoint [21]. On the basis of this trial, it was initially hypothesised a withdrawal proportion from allocated treatment within 3 months (primary study endpoint) of 25% in the group treated with clozapine plus haloperidol (control group). Moreover, it was hypothesised that the augmentation with aripiprazole (experimental group) would show a clinically significant advantage by producing a withdrawal proportion of 10%. A sample size of 194 patients (97 in each group) was chosen since it achieves 80% power to detect a difference of 15% between the two withdrawal proportions. The test statistic used was the two-sided Z test with pooled variance. The significance level of the test was targeted at 5%. Having assumed that 10% of the participants could be lost within 3 months, or could not provide valid data at month 3, the target total sample size for CHAT was 216 (= 194/0.90) patients in order to obtain 194 evaluable subjects [39,40].

Having considered the possibility that the target sample size could not be reached, we anticipated that the total sample size at the end of the enrolment period would be around 100 patients. With such a total sample size, CHAT achieves 80% power to detect a difference of 20% between the two withdrawal proportions (25% in the group treated with clozapine plus haloperidol versus 5% in the group treated with clozapine plus aripiprazole).

### Random Allocation Procedure

Patients were randomly assigned to one of the two treatment groups with an equal probability of assignment to each treatment (allocation ratio 1:1). A centralised randomization procedure was employed. The trial biostatistician prepared the sequence of treatments randomly permuted in blocks of constant size. The site investigators did not know the block size. The allocation was stratified by living condition (residential facility versus all the other living conditions) because in patients with resistant schizophrenia this hard variable may be considered a proxy of severity of illness. Recruiting physicians were asked to contact an operator at the World Health Organisation Collaborative Centre of the University of Verona. The operator had access to a computerised system that provides, after information on the enrolled participant was entered, the patient's identification number (ID) and the allocated treatment. The operator had not access to the randomisation lists. This procedure of randomisation was developed to fully conceal treatment allocation [41].

### Statistical consideration

The statistical analysis will be masked, i.e. the trial biostatistician will be blinded to the treatment groups until the analysis has been completed. Moreover, the trial biostatistician will not be involved in determining patients' eligibility, in administering the treatment, in measuring the outcomes or in entering data.

Two data locks will occur during the study. The first one will happen 3 months after the end of the enrolment period, when the information on the primary endpoint and on the short-term secondary endpoints will be available for all the participants. The second one will happen at the end of the study (12 months after the end of the enrolment period), when information on the long-term secondary endpoints will be available for all participants. Accordingly, two data analyses will be performed on an intention-to-treat (ITT) basis. All randomised participants who will receive at least one dose of the investigational drugs will be included in the ITT analysis. The outcomes of patients included in the non-randomized cohort will be presented descriptively. No formal statistical analysis has been planned to compare the randomized participants and the eligible, non-randomized patient cohort.

### Analysis of the Primary Outcome

In the randomized cohort, the proportion of patients withdrawing from the assigned treatment within 3 months will be compared between the two groups of treatment through the chi-square test. Additionally, we will calculate risk ratios and their 95% confidence intervals to corroborate the main analysis. A multivariate analysis (secondary analysis) will be performed through a Poisson regression model with a robust error variance, given that this procedure allows to estimate relative risks directly [42].

## Status of the trial and expected achievements

The recruitment phase started on September 1^st ^2006 and finished on December 31^st ^2008. During this period, 38 clinical sites across Italy actively participated in the study and recruited a total of 106 patients. This means that, despite the planned sample size of 216 patients has not been achieved, CHAT is the largest randomised study conducted so far in Western countries on this topic. Data collection, study monitoring and data management are performed by the coordinating centre (University of Verona). All study data are entered in a computerised database and stored by the World Health Organisation Collaborative Centre of the University of Verona. The person entering the data is not involved in determining patients' eligibility, administering treatment, or determining outcome. The correctness and consistency of the data is ensured by the double-entry technique and by a set of electronic and manual edit checks. The consistency of the data between the recruitment and follow-up forms and the computerised database are routinely verified. After each of the two data locks, masked data will be transferred to the Unit of Epidemiology and Medical Statistics of the University of Verona for statistical analysis.

The main achievements of this ongoing projects include the following: (a) the implementation of the CHAT protocol provides evidence that it is possible to create a network of community psychiatric services that accept the idea of using their everyday clinical practice to produce randomised knowledge; (b) the possibility of producing knowledge from the practice of medicine does not necessarily require huge financial support, as long as a pragmatic attitude to the evaluation of competitive treatment strategies is adopted; (c) the multicentre design, nested into everyday clinical practice, has been creating a situation where investigators simultaneously act both as physicians and researchers; (d) the pragmatic attitude employed allowed to randomly allocate considerable number of individuals [43]; (e) the current project, by generating evidence on whether it is clinically useful to combine clozapine with aripiprazole rather than with haloperidol, is expected to provide physicians with a solid evidence base to be directly applied in the routine care of patients with schizophrenia.

## Competing interests

The authors declare that they have no competing interests.

## Authors' contributions

CB, AC, SA, MN, MT participated in the conception and design of the trial. SA is the trial statistician and he made substantial contributions to the study design. AC, AV, FP, EE, SM helped manage the trial, collect reports and design data extraction sheets. PA^4^, FB^5^, RB^6^, DB^7^, GB^8^, BB^9^, AB^10^, RB^9^, FB^11^, MB^5^, AB^12^, PB^13^, MC^14^, LC^10^, LC^15^, GC^15^, PC^16^, BDS^4^, MDF^17^, GDL^18^, WDM^10^, GD^19^, AEr^20^, LF^3^, FF^21^, AF^22^, NF^23^, VFP^24^, MF^20^, FG^25^, NG^26^, AG^19^, GG^17^, LG^9^, NG^20^, LG^23^, GG^27^, FL^12^, EL^25^, CL^26^, FM^28^, LM^21^, LM^29^, AM^30^, MCM^31^, AM^14^, MM, GM^32^, MM^3^, DM^22^, SN^8^, FN^33^, GO^13^, AMP^29^, SP^23^, MP^16^, EP^34^, CP^32^, RP^35^, RQ^36^, LRi^6^, FR^29^, RR^33^, PR^11^, SR^19^, MR^31^, GRi^6^, GR^30^, AS^25^, CS^36^, GS^23^, AS^19^, FS^34^, SS^4^, TS^36^, AS^18^, SS^25^, GT^9^, AT^35^, RT^17^, RT^23^, SZ^13 ^participated in enrolling patient (details on superscripts are fully reported in the front page of the manuscript). MN drafted the manuscript. CB and AC critically reviewed the manuscript. All authors saw and approved the final version of the manuscript. The corresponding author had full access to all the data in the study and had final responsibility for the decision to submit for publication.

## Supplementary Material

Additional File 1 **CHAT inclusion and exclusion criteria (both for the randomised and for the observational cohort)**. The data provided represent the trial inclusion and exclusion criteria. Click here for file
