# Supplementary material for: Rationale and design of an independent randomised controlled trial evaluating the effectiveness of aripiprazole or haloperidol in combination with clozapine for treatment-resistant schizophrenia
Source: Trials. 2009 May 15;10:31. doi: 10.1186/1745-6215-10-31 (PMC2689216; doi:10.1186/1745-6215-10-31)
Supplement: Additional File 1 — CHAT inclusion and exclusion criteria (both for the randomised and for the observational cohort). The data provided represent the trial inclusion and exclusion criteria. [file 1745-6215-10-31-S1.ppt]

## Slide 1
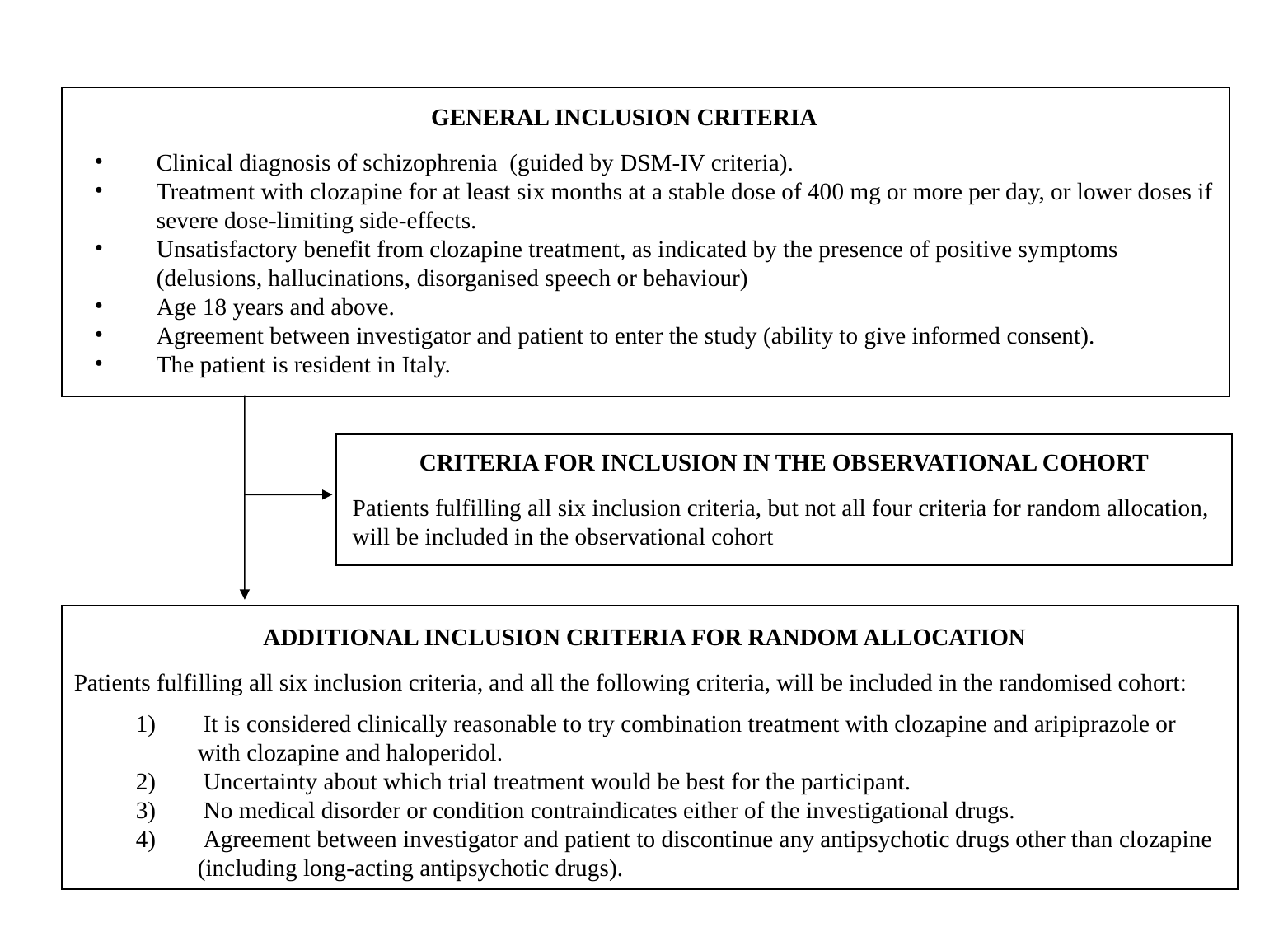

GENERAL INCLUSION CRITERIA
Clinical diagnosis of schizophrenia (guided by DSM-IV criteria).
Treatment with clozapine for at least six months at a stable dose of 400 mg or more per day, or lower doses if severe dose-limiting side-effects.
Unsatisfactory benefit from clozapine treatment, as indicated by the presence of positive symptoms (delusions, hallucinations, disorganised speech or behaviour)
Age 18 years and above.
Agreement between investigator and patient to enter the study (ability to give informed consent).
The patient is resident in Italy.
CRITERIA FOR INCLUSION IN THE OBSERVATIONAL COHORT
Patients fulfilling all six inclusion criteria, but not all four criteria for random allocation, will be included in the observational cohort
ADDITIONAL INCLUSION CRITERIA FOR RANDOM ALLOCATION
Patients fulfilling all six inclusion criteria, and all the following criteria, will be included in the randomised cohort:
 It is considered clinically reasonable to try combination treatment with clozapine and aripiprazole or with clozapine and haloperidol.
 Uncertainty about which trial treatment would be best for the participant.
 No medical disorder or condition contraindicates either of the investigational drugs.
 Agreement between investigator and patient to discontinue any antipsychotic drugs other than clozapine (including long-acting antipsychotic drugs).
